# Exploring the Power of Deep Learning: Fine-Tuned Vision Transformer for Accurate and Efficient Brain Tumor Detection in MRI Scans

**DOI:** 10.3390/diagnostics13122094

**Published:** 2023-06-16

**Authors:** Abdullah A. Asiri, Ahmad Shaf, Tariq Ali, Unza Shakeel, Muhammad Irfan, Khlood M. Mehdar, Hanan Talal Halawani, Ali H. Alghamdi, Abdullah Fahad A. Alshamrani, Samar M. Alqhtani

**Affiliations:** 1 Radiological Sciences Department, College of Applied Medical Sciences, Najran University, Najran 61441, Saudi Arabia; aaalasmy@nu.edu.sa; 2 Department of Computer Science, COMSATS University Islamabad, Sahiwal Campus, Sahiwal 57000, Pakistan; tariqali@cuisahiwal.edu.pk; 3 Department of Biosciences, COMSATS University Islamabad, Sahiwal Campus, Sahiwal 57000, Pakistan; 4 Electrical Engineering Department, College of Engineering, Najran University, Najran 61441, Saudi Arabia; miditta@nu.edu.sa; 5 Anatomy Department, Medicine College, Najran University, Najran 61441, Saudi Arabia; kmmehdar@nu.edu.sa; 6 Computer Science Department, College of Computer Science and Information Systems, Najran University, Najran 61441, Saudi Arabia; hthalawani@nu.edu.sa; 7 Department of Radiological Sciences, Faculty of Applied Medical Sciences, The University of Tabuk, Tabuk 47512, Saudi Arabia; ah.alghamdi@ut.edu.sa; 8 Department of Diagnostic Radiology Technology, College of Applied Medical Sciences, Taibah University, Madinah 41477, Saudi Arabia; ashamrani@taibahu.edu.sa; 9 Department of Information Systems, College of Computer Science and Information Systems, Najran University, Najran 61441, Saudi Arabia; smalqhtani@nu.edu.sa

**Keywords:** brain tumor, vision transformer, healthcare, diagnosis

## Abstract

A brain tumor is a significant health concern that directly or indirectly affects thousands of people worldwide. The early and accurate detection of brain tumors is vital to the successful treatment of brain tumors and the improved quality of life of the patient. There are several imaging techniques used for brain tumor detection. Among these techniques, the most common are MRI and CT scans. To overcome the limitations associated with these traditional techniques, computer-aided analysis of brain images has gained attention in recent years as a promising approach for accurate and reliable brain tumor detection. In this study, we proposed a fine-tuned vision transformer model that uses advanced image processing and deep learning techniques to accurately identify the presence of brain tumors in the input data images. The proposed model FT-ViT involves several stages, including the processing of data, patch processing, concatenation, feature selection and learning, and fine tuning. Upon training the model on the CE-MRI dataset containing 5712 brain tumor images, the model could accurately identify the tumors. The FT-Vit model achieved an accuracy of 98.13%. The proposed method offers high accuracy and can significantly reduce the workload of radiologists, making it a practical approach in medical science. However, further research can be conducted to diagnose more complex and rare types of tumors with more accuracy and reliability.

## 1. Introduction

An abnormal/uncontrolled cellular growth within the brain and surrounding tissues is an intracranial neoplasm or brain tumor. This uncontrolled benign or malignant growth can originate either from the brain cells, a condition known as a primary brain tumor, or from surrounding metastasized cells/tissues causing metastases (secondary brain tumor). According to the CBTRUS statistical report, more than 700,000 people (about half the population of Hawaii) currently suffer from a primary brain tumor or intracranial neoplasm. Approximately 84,000 new cases are reported and diagnosed each year. Brain tumors may occur in people of all ages but are most diagnosed in children and older adults [1,2]. Headaches, cognitive changes, and seizures are brain tumors’ most common symptoms. Headaches, being the most frequent symptom, are usually described as dull or sharp. Seizures are described as convulsions, loss of consciousness, and muscle twitching. Patients with brain tumors also experience cognitive changes such as memory, concentration, and reasoning difficulty. Other symptoms of brain tumors include poor vision or hearing, weakness of the limbs, and difficulty with speech or language [3]. Physicians use several factors to categorize and detect brain tumors. These factors include location, size, and distinct imaging features [4]. Meningioma, noncancerous brain tumors, arise from the brain’s membranous tissues. Gliomas tumors originate from glial cells, while glioblastomas arise from the brain. Both gliomas and glioblastomas are cancerous brain tumors [5]. Another type of tumor (pituitary) originates in an important gland within the brain (pituitary gland). The pituitary gland is responsible for the regulation of other glands in the body. Physicians can accurately diagnose and treat brain tumors [6] based on these unique characteristics of brain tumors.

Ultra-violet radiation, chemicals, and surgical procedures are standard treatment methods for brain tumors. Brain tumors negatively impact both patients and their families. Hence, early detection is crucial for improved prognosis [7]. There are several imaging techniques used for brain tumor detection. Among these techniques, the most common is MRI which stands for magnetic resonance imaging. MRI detects brain tumors using magnetic fields and radio waves [8]. Another technique is computed tomography, also known as a CT scan. This technique uses X-rays to detect brain tumors. Brain tumors can also be detected through PET, known as positron emission tomography. This imaging technique works by injecting a radioactive substance into the blood. Among the surgical procedures, a biopsy is the most effective method for brain tumor detection. A small piece of the tumor is examined under a microscope to determine the type of brain tumor. These imaging techniques are effective for brain tumor detection [9]. However, these conventional methods have several limitations as well. These imaging techniques are expensive and time-consuming. It can be difficult for patients who require frequent follow-up scans [10]. Moreover, the accuracy of these imaging techniques can be compromised by the size and location of the tumor and the presence of surrounding tissues. There are chances of false outcomes represented by the confusion matrix of the model. This can lead to incorrect diagnosis and delayed treatment [11].

Moreover, the accuracy of these imaging techniques can be compromised by the size and location of the tumor and the presence of surrounding tissues. There are chances of false outcomes represented by the confusion matrix of the model. This can lead to incorrect diagnosis and delayed treatment. Several machine and deep learning models are also used to detect brain tumors to avoid the problems mentioned above. Algorithms are used to train the model using input data of patients’ MRI scans and images. The model then predicts the existence or occurrence of brain tumors based on the image provided [12]. A supervised learning algorithm (SVM) can efficiently detect brain tumors through classification and segmentation modelling. The ensemble learning algorithm that can conduct both categorical analysis (classification) and predictive analysis (regression) for tumor detection is known as random forest (RF). CNN, conventional neural network, a deep learning algorithm, is also used for brain tumor detection. CNN performs an image classification task. This algorithm extracts features from brain images, classifies them based on those features, and detects brain tumors [13].

In [14], the researchers utilized a pretrained (CNN) model, specifically known as the VGG-16 model, as a foundation and modified it to identify several types of brain tumors. The dataset used for this study was the CE-MRI dataset, which comprises MRI images of brain tumors classified into four distinct categories. The dataset has a sample of 233 patients’ MRI scans and images (a total of 3064), which were used first to train the model and then validate the output generated through the model. The study’s findings demonstrated that the proposed methodology was adequate, with an overall accuracy of 94.82% in categorizing brain tumor images into the four distinct groups. In [15], the authors trained an (CNN) approach on data from one specific group. They tested it on data from two other groups to determine whether the algorithm’s performance would be affected by differences in data acquisition and processing across institutions. The authors used the TCIA dataset. The results showed that CNN’s performance was significantly better when trained on image data or scans from the same grouping/institution than when trained on data from one institution and tested on data from another institution. Specifically, the DSC score for the institution was reported as 0.72 ± 0.17 and 0.76 ± 0.12. The DSC score for different institutions was reported as 0.68 ± 0.19 and 0.59 ± 0.19. The study [16] presents an approach based on deep learning to classify tumors through MRI data.

The authors propose a deep CNN architecture called the BraTS-Net, which incorporates both 2D and 3D convolutions and a combination of modules (most common are inception and residual) to improve performance. The authors used the BraTS 2013 dataset, which includes MRI images and scan data of patients with necrosis, edema, and low-grade gliomas (LGG). In the proposed work, compared with SVMs, RFs concluded that the BraTS-Net achieved significantly better results, with an overall DSC score of 0.88. However, the study does not evaluate the generalizability of CNN to other datasets or imaging modalities. In [17], the authors developed a CNN architecture consisting of three convolutional layers along with two fully connected layers and trained it using a dataset of MRI scans from patients diagnosed with tumors using the BAT algorithm. The authors used the BraTS 2015 dataset, which contains MRI image data of glioma patients. The model achieved an accuracy of 92% with a specificity of 87% and a sensitivity of 90%. However, the authors need to compare their model’s performance with human experts, which could provide insight into the model’s limitations and potential areas for improvement. The authors developed a deep neural network framework called DeepSeg [18], which is designed to segment brain tumors into four categories: edema, tumor (type: nonenhancing), tumor (type: enhancing), and necrosis. The authors trained and tested their model on the BraTS 2019 dataset, which contains images and scans of patients with gliomas. They concluded the results using the U-Net, DeepMedic, and BraTS baseline methods and found that their model achieves better results, with an average DSC score of 0.814, sensitivity score of 0.783, and specificity score of 0.999. However, the study lacks other performance evaluation metrics such as confusion metrics and F1 scores.

The authors used a combination of YOLOv2, NSGA, LDA, SVM, k-NN, and Inception-v3 to perform tumor detection and segmentation using MRI scans and images [19]. The authors trained their model on the (BraTS 2018–2020) dataset, which contains MRI data of patients with gliomas, HGG, and LGG. The study results show that the strategy mentioned above achieves satisfactory performance in successfully detecting and segmenting brain tumors. The model achieved a classification accuracy of 98% to 99% for BraTS (2018–2020). However, the authors only evaluated their model on one dataset, and how it would perform on other datasets or with other types of brain tumors remains to be seen. The authors did not provide specificity, confusion matrix, or DSC performance scores. In [20], a novel approach for efficient identification and segmentation of tumors using MRI image data was proposed. The authors improved the accuracy of the segmentation task by combining CNN with a transformer. The proposed Bitr-unit model has an architecture composed of two significant components: an encoder (CNN based) and a decoder (Transformer-based). To evaluate the outcome and working of their model, the authors used a publicly available dataset: BraTS 2021. They compared the results of their model with other methods, including Unet++, UNet, and BRATS-2. The results showed that the working model, the Bitr-unit model, achieved better performance in terms of segmentation, with an overall DSC score of 0.908 for the whole tumor. The authors also describe the FT-ViT, making it easy for other researchers to replicate and build upon their work. However, the detailed requirements for the FT-ViT needed to be included, which could be a limiting factor for real-time application design. In [21], a new architecture was designed for semantic brain tumor segmentation tasks. The authors built on the Swin Transformer architecture, which proved itself as an effective model for natural image classification tasks, and modified it to handle medical image segmentation tasks. The authors tested the model on the BraTS 2021 dataset, a benchmark for identifying brain tumors. They compare their method with several previous methods and reported that it achieves top-notch performance in terms of segmentation accuracy while being faster and more memory efficient. The DSC score for the whole tumor was reported to be 0.933. However, the authors only evaluated their method on one dataset, which may not represent the full range of variations in medical images. In study [22], the authors propose a novel architecture called the Volumetric Transformer Network (VTN), which extends the standard transformer architecture to handle 3D volumetric data. The authors evaluated this strategy on two publicly available datasets: the Brain Tumor Segmentation (BraTS) 2021 dataset and the Head and Neck Auto-segmentation Challenge (Hecktor) dataset.

Compared to other ML approaches, the authors reported that VTN achieves competitive results regarding segmentation accuracy while being faster and more memory efficient. The results showed a DSC score of 91.20% for the whole tumor. However, one profound limitation is that the essential detailed analysis of the interpretability of the Transformer network needed to be included. It needs to be clarified how the Transformer network is making its segmentation decisions, which could limit the clinical applicability of the method. In another study [23], the authors addressed the challenges of multimodal brain tumor classification and segmentation problems (MRI-based), such as the heterogeneity and variability of the tumor tissue, using a Transformer network that can capture the complexities between different modalities. The proposed method, Transbts, consists of two stages. In the preprocessing stage, the input MRI images are preprocessed and transformed into a multi-scale feature map. The Transformer network is used to segment the feature map in the segmentation stage. The authors compare their method with several ML methods (BRATS 2019–2020). The results showed a DSC score of 90% for the whole tumor (BRATS 2019) and 90.09% for the whole tumor (BRATS 2020). One of the strengths of this study is that the authors thoroughly evaluate their method on two large and publicly available datasets, which makes the results more reliable and generalizable. However, the authors must report the computational resources required for training and inference, which could be a significant barrier to adopting and practicing the method as a clinically approved method.

These machine and deep learning algorithms are useful for detecting brain tumors but are also limited. The output of these algorithms relies on the quality and quantity of the data. These algorithms may not effectively identify complex classes of tumors that are exceptional and rare types of brain tumors. The feature selection, model architecture, and other parameters can also impact the accuracy of these algorithms [24]. These drawbacks highlighted the need for a more accurate and efficient technique for brain tumor detection. A deep learning method called Vision Transformer is a promising solution to overcome the current limitations. Vision Transformer with a neural network framework is a unique algorithm for computer vision tasks [25]. The ViT model detects complex tumors by breaking input data into chunks of images and applying self-attention mechanisms. This model learns the difference between healthy and cancerous tissue in the training process by minimizing a loss of function.

The output is generated as a probability map during the testing process, giving information about brain regions with tumors [26]. This study addresses the question: can a vision transformer model accurately detect brain tumors in medical imaging? The hypothesis statement says vision transformer models trained on MRI scans and images can achieve high accuracy in detecting brain tumors, outperforming existing methods for brain tumors. Unlike the traditional methods, which use convolutions to analyze 3D relationships between pixels in the brain tumor images, ViT learns global relationships between the image features using self-attention mechanisms. Vision Transformer automatically extracts features from brain tumor images and detects tumors based on those features. Several brain tumor datasets containing extensive medical images and related clinical data are used to train and test multiple machine and deep learning algorithms. With the help of these datasets, researchers and clinicians can detect, segment, and diagnose brain tumors. A few of these brain tumor datasets are: the datasets consisting of MRI scans and images of the most common yet aggressive form of tumor (glioblastoma), known as BraTS (The Brain Tumor Segmentation Challenge Dataset). This dataset has the MRI scans and related clinical data of more than 200 patient cases.

BraTS is widely used for detecting, classifying, and segmentation problems related to brain tumors [27]. Low-grade glioma, LGG, has a better prognosis than other brain tumors because it grows slowly. The dataset containing MRI scans and images of patients suffering from LGG is known as LGG-1p19qDeletion Dataset. It is widely used for the classification and segmentation of LGG brain tumors. Other datasets have MRI scans and images of glioblastoma with some modifications (T1 and T2 weighted and FLAIR scans) [28]. TCGA-GBM is another dataset containing MRI images and genomic data of glioblastoma.

Along with the genomic data, MRI scans, and other patient clinical data, the CPTAC-GBM dataset also contains proteomic data of patients with glioblastoma [29,30]. All the datasets mentioned above are used to train various machine and deep learning models. These models then test input data to detect, classify, and segment brain tumors.

The research aims to:

- Develop and evaluate an accurate model for efficiently identifying brain tumors from images.
- Fine-tune the Vision Transformer (ViT) model to achieve higher accuracy and faster inference times for tumor detection.
- Explore the use of transfer learning, data augmentation, and other techniques to improve the outcome and efficiency of the model.
- Provide a reliable and automated tool for early identification and prognosis of brain tumors, reducing the high detection costs and improving the healthcare industry.

The rest of the paper is organized as follows: Section 2 describes the related work, Section 3 explains the detail of materials and methodology we have adopted for the proposed work, Section 4 explains the detailed results section, and finally concludes the paper in the conclusion section.

## 2. Materials and Methods

### 2.1. Dataset

In this study, we used data that consisted of medical scans and images of the brain, specifically MRI scans. The images are preprocessed and labeled to indicate whether they contain a brain tumor. The dataset is large, with thousands of images, and is representative of the population of patients who undergo brain tumor diagnosis. The images in the dataset are typically annotated to indicate the presence and location of tumors.

This annotation can be performed manually by radiologists or other medical experts or automated using computer algorithms. The data (MRI Scans) used to conduct this study are an open-access dataset (available at https://www.kaggle.com/datasets/masoudnickparvar/brain-tumor-mri-dataset?select=Training, accessed on: 3 March 2023) consisting of 2D scans and images with significant slice gaps [31]. This dataset was obtained from various hospitals in China between 2005 and 2020 and included four categories of tumors: glioma, meningioma, pituitary, and no tumor. Gliomas arise from glial tissues surrounded by neurons and are a common type of brain tumor, while meningiomas develop from the surrounding meninges tissues. Pituitary tumors result from abnormal growth in a gland at the back of the nose (pituitary gland). The image dimensions of the dataset are presented as 512 × 512 pixels.

### 2.2. How ViT Works

The basic idea behind a vision transformer is to break down an image into small patches and feed them through a neural network. Each patch is processed individually, and the output from each patch is combined to allow the network to learn the overall structure and features of the image. The input image is typically a scan or image (MRI) of the patient’s brain for efficient identification of complex brain types of brain tumors. The vision transformer breaks down the MRI into small patches and processes each patch individually. A concatenation process where the outcome of each patch is summed up occurs to create a representation of the entire image. Once the vision transformer has processed raw input data, it can be used to segment and classify the image as either containing a brain tumor or not. This is typically performed using a binary classification algorithm that takes the output from the vision transformer as input. Using a vision transformer, the whole working model can process, decode, and learn about the complex features of each type of tumor, allowing it to detect them accurately in new images.

### 2.3. Fine-Tuned Vision Transformer Architecture

**Figure 1:**
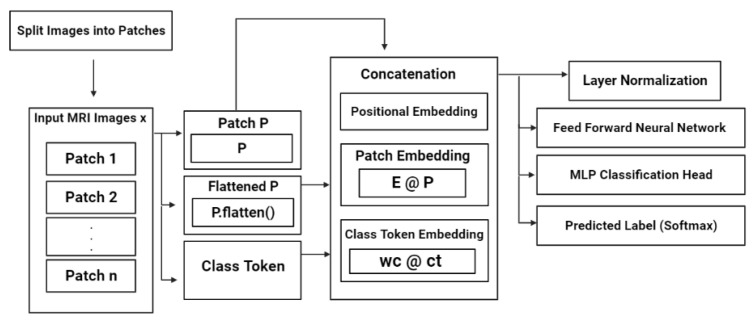
Overview of the FT-ViT architecture displaying the sequential steps involved in processing input images. The process includes patch splitting, patch flattening, concatenation of embeddings (positional, patch, and class token), layer normalization, feed-forward neural network (FFNN), MLP Head, and Softmax function.

In the Vision Transformer (ViT) architecture for brain tumor detection [31], the backend algorithm splits the input MRI scans and images into patches. This step is necessary because the input image may be too large to be processed by the model directly. The splitting of the input image is achieved by dividing the image into a regular grid of nonoverlapping patches. Each patch is then linearly flattened into a single long vector. The flattened patches are then fed into the model for further processing as shown in Figure 1.

Mathematically, the matrix X denotes the input MRI image and scan data. The splitting of the input scan into patches can be expressed as follows:

# Input MRI image X of size V × Z × C

# Patch size P × P × C

# Stride S

$$N\_\mathrm{row}\;=\;\mathrm{floor}\left(\left(Z\;-\;P\right)/u\right)+1$$

$$N\_\mathrm{col}\;=\;\mathrm{floor}\left(\left(O\;-\;P\right)/u\right)+1$$

patches =[]

# Iterate over N_row

# Iterate over N_col

$$\;\mathrm{patch}\;=\;X\left[j\times Q:j\times S+R,i\times Q:i\times S+P,∶\right]$$

$$\mathrm{patch}=\mathrm{patch}.\mathrm{flatten}\left(\;\right)$$

$$\mathrm{patches}.\mathrm{append}\left(\mathrm{patch}\right)$$

**Figure 2:**
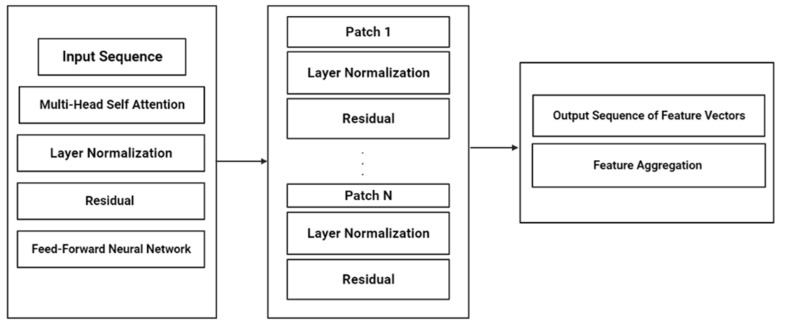
Feed-Forward Neural Network (FFNN) with Residual Output Sequence and Feature Aggregation.

The resulting set of flattened patches is then fed into the next stage of the ViT model for further processing as shown in Figure 2. The next step after splitting the input MRI brain images into patches is to flatten the patches into a single long vector. This step is necessary to convert the coming patch data into a standardized format for further processing (self-attention module). Mathematically, if a matrix represents a patch P-sized P × P × C (here, C represents the number of image channels), the flattening of the patch can be expressed as follows:

patch_flat = P.flatten()

This operation converts the P × P × C matrix into a 1D vector of length P × P × C. After flattening all the patches, the resulting set of flattened patches is fed into the ViT model’s self-attention mechanism for further processing. After flattening the patches, the next step in the Vision Transformer (ViT) architecture for brain tumor detection is to produce lower-dimensional linear embeddings from the flattened patches. This step is accomplished by applying two linear transformations to the flattened patches: the patch embedding projection and the class token projection. The patch embedding projection linearly maps the flattened patch vector to a lower-dimensional feature vector. This projection matrix is a learnable parameter in the ViT model and is denoted as matrix E-sized (D, P × P × C). Here D is the dimensionality of the patch embedding. Mathematically, this operation can be expressed as follows:

patch_embedding = E @ patch_flat

where @ represents the matrix multiplication operation.

$$X\;=\left[x1,x2,\ldots ,xN\right]$$

X_emb= X ∗ E

The second linear transformation is the class token projection, which linearly maps a learnable class token vector to a lower-dimensional feature vector. The class token vector is a learnable parameter in the ViT model and works as a fixed-length presentation of the entire image. Mathematically, this operation can be expressed as follows:

class_token_embedding = Wc @ class_token

where “Wc” represents a matrix (Learnable Parameter) sized (D, D) and class_token is a learnable D-dimensional vector. The patch embeddings and class token embedding are then concatenated and fed into the transformer encoder for further processing. After producing the lower-dimensional linear embeddings from the flattened patches and class token, the next step in the Vision Transformer (ViT) architecture for brain tumor detection is to add positional embeddings to the concatenated embeddings. This step is necessary to provide the ViT model with information about the spatial position of each patch within the original image. The positional embeddings are added to the concatenated embeddings using the same matrix as Wc-sized (D, L). D represents the dimensionality of the patch embeddings, whereas L denotes the maximum length of the sequence of the concatenated embeddings. The resulting matrix is then passed through a layer normalization (LN) operation. Mathematically, this operation can be expressed as follows:

$$\mathrm{positional}\_\mathrm{embeddings}\;=\;P\;@\;\left[\mathrm{patch}\_\mathrm{embedding};\;\mathrm{class}\_\mathrm{token}\_\mathrm{embedding}\right]$$

$$\mathrm{embedding}\;=\left[\mathrm{patch}\_\mathrm{embedding};\;\mathrm{class}\_\mathrm{token}\_\mathrm{embedding};\;\mathrm{positional}\_\mathrm{embeddings}\right]$$

$$\mathrm{embedding}\_\mathrm{normalized}\;=\;\mathrm{LN}\;\left(\mathrm{embedding}\right)$$

“@” represents the matrix multiplication operation, “;” represents the concatenation operation along the second dimension, and [q; r] denote the concatenation of tensors q and r along the second dimension. The resulting embedding normalized tensor has shape (L, D) and contains the patch embeddings, class token embedding, and positional embeddings. The flattened patches are enhanced by including positional embeddings, and the sequence that comes as output is used further as an input for a transformer encoder that comprises multiple layers. Each layer of the transformer encoder comprises an MSA module and an FFN module. The MSA module utilizes scaled dot-product attention, calculates the whole sum of all the weighted values in the sequence, and provides an output of the MSA module by processing it through a residual connection and then passed to an operation (layer normalization) before being passed to the FFN module. Within the transformer encoder, the FFN module comprises two linear layers and is accompanied by a ReLU activation function. The resulting output from the FFN module is then routed through a series of repeated LN steps before being processed by the next layer. Then, the final layer of the transformer encoder yields a sequence of feature vectors, each representing a distinct patch of the input image. Then, a unified feature is generated by assembling the previous feature vectors representing the complete image [32].

Z^l = SelfAttnLNX_emb^l+ X_emb^l

H^l = FFNLNZ^l+ Z^l

The following equations define the MSA and FFN modules:

**The process of the MSA module:** we have a sequence of input vectors, {p_1, p_2, …, p_n}. The MSA module calculates the output as follows:

Firstly, the input vectors are converted into query, key, and value vectors:

$$\mathrm{Que}\;=\;V\;*\;H\_\mathrm{Que},\mathrm{Ke}\;=\;V\;*\;H\_\mathrm{Ke},V{}^{\prime }\;=\;V\;*\;H\_V$$

V is the input sequence, and H_Q, H_K, and H_V are trainable weight matrices. The dot-product attention scores are calculated using the following equation:

AttentionQue,Ke,V′= softmaxQue ∗ Ke ^ T/sqrtdim_k ∗ V′

dim_k denotes the key vector’s dimensionality. The MSA module’s output is the weighted sum of the values using the attention scores:

$$\mathrm{Output}\;=\;\mathrm{scores}\_\mathrm{attention}\left(\mathrm{Que},\mathrm{Ke},V{}^{\prime }\right)\;*\;H\_O$$

where H_O is a learnable weight matrix.

**Feed-Forward Neural Network Module:** we have an input vector x. The FFN module generates the output as follows:

$$\mathrm{FFN}\left(x\right)=\;\max\left(0,x\;*\;c\_1+\;k\_1\right)\;*\;c\_2+\;k\_2$$

where c_1, c_2, k_1, and k_2 are trainable weight matrices and bias vectors. Moreover, ax (0, x) refers to the ReLU activation function.

$$z=\;*\left(\left[z1,z2,\ldots ,zN\right]\right)$$

$$y\;=\;\mathrm{Softmax}\left(\mathrm{MLP}\left(z\right)\right)$$

During training, the ViT model is pretrained on a large dataset using a contrastive loss function, which encourages similar representations for similar images and different representations for dissimilar images. The pretrained model is then transferred to a fine-tuning process. The target of classification is achieved via a loss function (cross-entropy)

L_CE=−\sum_i=1^c ∗ w_i \log\hatw_i+1−w_i \log(1−\hatw_i

The ground truth label is denoted by w_i for the ith image, \hat{w_i} is the output (prediction based) for the ith image, and “c” is the number of images in the training dataset.

**Model Ensembling:** To improve the classification performance, multiple ViT models are trained, and their Softmax outputs are ensembled using a weighted average:

$$y\_\mathrm{ensemble}=\;\mathrm{Softmax}\left(w1\;*\;y1+\;w2\;*\;y2+\ldots +\;\mathrm{wn}\;*\;yn\right)$$

y_i is the output probability distribution of the i-th ViT model, and w_i is the weight assigned to the i-th model.

## 3. Results

The study aimed to develop an efficient approach for the detection and classification of brain tumors using Vision Transformers (FT-ViT). The objective was to address the critical need for accurate and reliable identification of brain tumor types to support medical diagnosis and treatment planning. By leveraging the power of Vision Transformers, the study aimed to achieve high accuracy in classifying different brain tumor classes, including pituitary tumor, no tumor, meningioma tumor, and glioma tumor. The study focused on evaluating the performance of the proposed FT-ViT model through comprehensive analysis and comparison with existing approaches, contributing to the advancement of brain tumor detection in the field of medical imaging. The dataset used in this study comprised 5712 MRI scans, each belonging to one of four classes: pituitary tumor, no tumor, meningioma tumor, and glioma tumor. The dataset was split using an 80:20 ratio for training and validation, respectively. The training set had 4569 images, while the validation set contained about 1143. The FT-ViT (Vision Transformer) model was employed, and the input images were preprocessed using the ImageDataGenerator function from the TensorFlow library, with the “tf.keras.applications.mobilenet_v2.preprocess_input” method.

**Figure 3:**
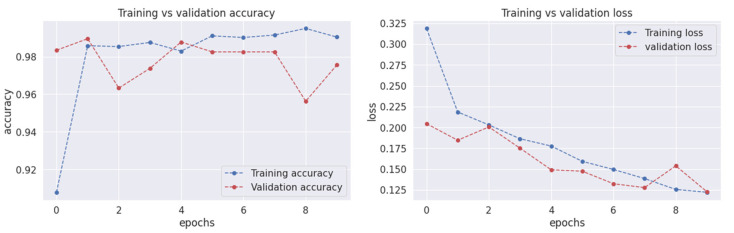
Training V/s Validation Accuracy and Loss Graphs.

The performance of the FT-ViT model was evaluated using various metrics, including accuracy, precision, recall, and F1 score, to assess its effectiveness in classifying brain tumor images. The model achieved an impressive validation accuracy of 98.13%, demonstrating its ability to accurately identify brain tumors. Additionally, training vs. validation accuracy and loss graphs were generated to visualize the model’s learning progress as shown in Figure 3. The training accuracy graph demonstrated a gradual improvement in accuracy over the training epochs, reaching an impressive value of 0.9813. The validation accuracy initially started at a high value but exhibited fluctuations during the training process. However, it eventually stabilized at a value of 0.97. The training and validation loss graphs followed similar trends, showing a decrease in loss over time.

**Figure 4:**
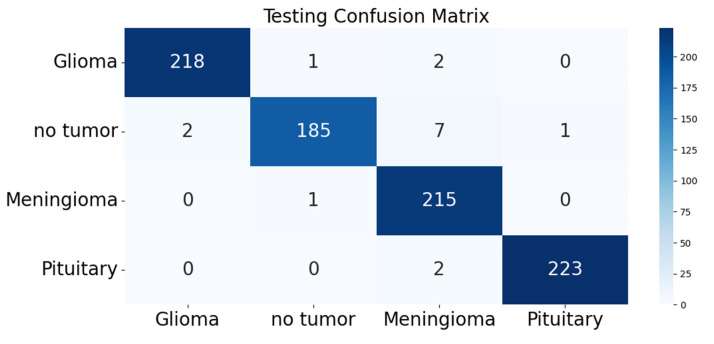
Confusion Matrix of FT-ViT.

**Figure 5:**
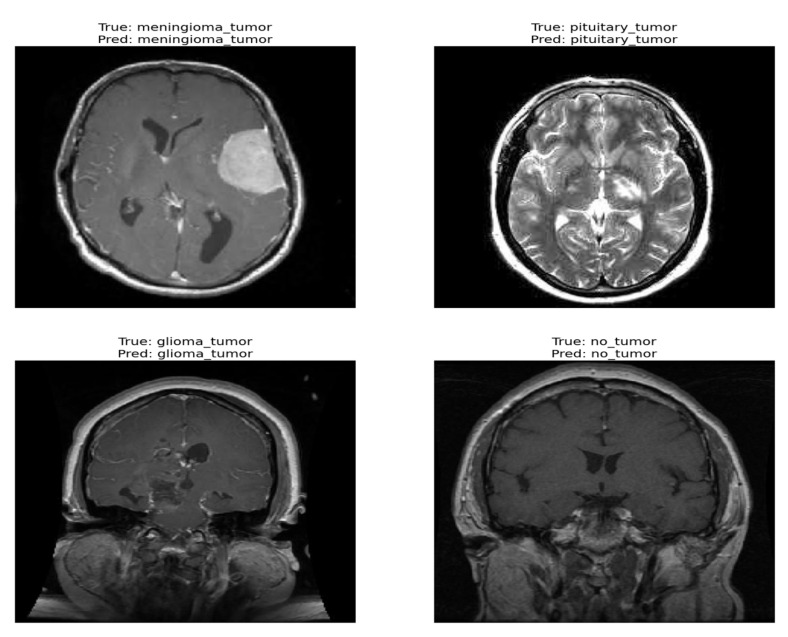
Correct classification result of proposed ViT model.

**Figure 6:**
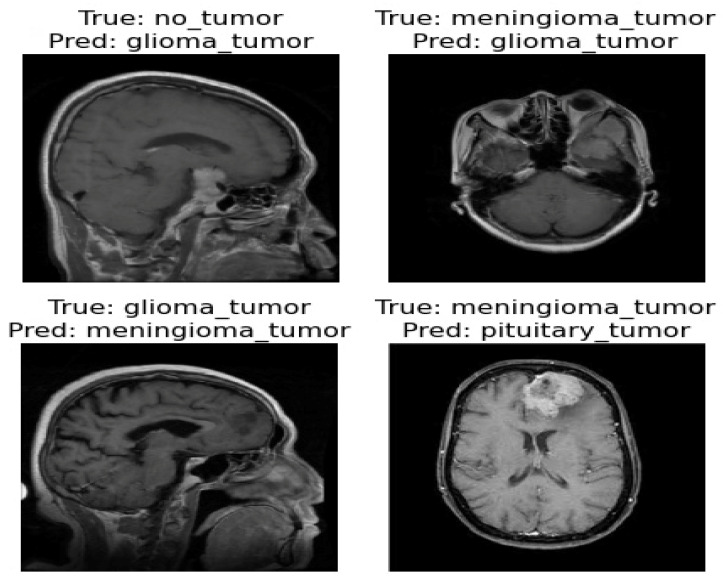
Incorrect classification result of proposed ViT model.

The confusion matrix was utilized to provide additional insights into the model’s performance by summarizing the correctly and incorrectly classified instances. For the validation set of 857 images, the model correctly identified 841 images, while 16 images were misclassified as shown in Figure 4. Furthermore, Figure 5 and Figure 6 present the classification outcomes of the proposed study, illustrating both accurate and inaccurate classifications.

The classification report provided detailed insights into the model’s performance for each tumor class as shown in Table 1. For the pituitary tumor class, the model achieved a precision of 1.00, recall of 0.99, and F1 score of 0.99, with the support of 225 images. In the no tumor class, the model achieved a precision of 0.95, recall of 1.00, and F1 score of 0.97, with the support of 195 images. The model’s performance for the meningioma tumor class resulted in a precision of 0.99, recall of 0.95, and F1 score of 0.97, with the support of 216 images. Lastly, for the glioma tumor class, the model achieved a precision of 0.99, recall of 0.99, and F1 score of 0.99, with the support of 221 images.

The overall accuracy of the model, as measured by the classification report, was 0.98, indicating its ability to correctly classify brain tumor images across all classes. The macro average precision, recall, and F1 score were also 0.98, further demonstrating the model’s consistent performance across the different tumor classes. The weighted average precision, recall, and F1 score were also 0.98, indicating that the model achieved balanced performance considering the dataset’s class distribution.

Figure 5 and Figure 6 present the visual results obtained from our FT-ViT model. These figures serve as compelling evidence of the model’s efficacy in accurately classifying different tumor types. As depicted in Figure 5, the proposed FT-ViT model exhibits remarkable performance, achieving an impressive accuracy of 98.13%. The high accuracy underscores the model’s capability to correctly identify tumor types with exceptional precision. However, there are instances where the FT-ViT model encounters challenges in accurately classifying tumor types. These instances are also reflected in the confusion matrix, which highlights the cases where the model misclassified tumor types. Figure 6 provides a visual representation of the results where the FT-ViT model encountered difficulties and failed to identify certain tumors accurately. The incorporation of both Figure 5 and Figure 6 allows us to comprehensively evaluate the FT-ViT model, highlighting its overall success in tumor classification, while also shedding light on areas for improvement.

These results demonstrate the credibility of the FT-ViT to be used to identify different brain tumors. The FT-ViT, being cost efficient, less time consuming, and more accurate, has the potential to improve the medical diagnosis procedures. Comparing the results of the FT-ViT (Vision Transformer) with other relevant studies, Table 2 demonstrates that our model has provided more reliable and efficient results in terms of accuracy. For instance, in study [33], the CNN-based approach was utilized, and it achieved the accuracy of 97% on 70% training dataset. In another study, [34], the GAN (generative adversarial networks) model was trained on a 60% training dataset, and it achieved an accuracy of 96.25%. The MANet approach [35] was used to achieve 97.71% accuracy on identifying brain tumors. Another approach based on GAN [36], achieved 96% accurate results in identifying tumors. Another BW-VGG19 model [37] maintained the accuracy score of 98%. Our FT-ViT (Vision Transformer Encoder) performed more efficiently than all the above-mentioned studies and identified 98.13% of the complex types of brain tumors accurately and reliably. The achievement in terms of more accurate results can help improve the tumor diagnosis and identification in the medical imaging industry.

## 4. Discussion

The results of this study demonstrate the effectiveness of the proposed approach, which utilizes the Vision Transformer (FT-ViT), for brain tumor detection based on MRI scans. The model achieved a high accuracy of 98.13% on the dataset, accurately identifying the four types of brain tumors: pituitary tumor, no tumor, meningioma tumor, and glioma tumor.

The ImageDataGenerator function from the TensorFlow library was used to preprocess the input images using the “tf.keras.applications.mobilenet_v2.preprocess_input” method. The output of the FT-ViT model was checked using the accuracy metric, which measures the percentage of correctly classified images among all the images. The model shows an impressive accuracy of 98.13% on the validation set. This demonstrates the superiority of using a vision transformer model for brain tumor detection compared to other studies. While accuracy is an important evaluation metric, it is not the only standard metric that should be considered when evaluating a model’s efficiency. Other metrics such as precision, recall, and F1 score were also considered, along with factors such as data classification report and model complexity, to ensure that the model is performing well in all aspects.

To present the results in a more compelling way, two graphs were generated. The training V/s validation accuracy graph demonstrates how well the FT-ViT performs during training as well as the testing process and what percentage of accuracy is achieved during both testing and training processes. The *y*-axis of this graph denotes accuracy, which measures the percentage of correctly classified images, while the *x*-axis of this graph represents the term epochs, which refers to the number of times the model has gone through the entire training dataset. The graph indicates that the model’s training accuracy started from a low value of 0.20 at the beginning of the training process and gradually improved over time. After eight epochs, the training accuracy reached an impressive value of 0.9813, indicating that the model is impressively learning the new features of the input images and classifying them.

On the other hand, the validation accuracy started from a high value of 0.98, indicating that the model is performing well on unseen data. However, the validation accuracy fluctuated during the training process, with a drop to 0.96 at the second epoch and another drop to 0.95 at the eighth epoch. These drops in validation accuracy could be due to over-fitting, where the model starts to memorize the training data instead of learning its general features. However, the validation accuracy improved after the eighth epoch and stabilized at a value of 0.97, proving that the proposed model is efficiently recognizing the complex features and generalizing to the unseen data. The training V/s validation loss graph shows a similar trend, where the training loss decreases gradually over time, showing that our model is well trained in learning the features of input data over time. On the other hand, the validation loss fluctuates during the training process but eventually stabilizes at a low value, indicating that the model is not over-fitting to the dataset used in the training process. Overall, the training V/s validation accuracy and loss graphs suggest that the proposed method is more reliable and accurate in detecting brain tumors from images, with high accuracy and low loss values. However, it is important to be cautious of overfitting and ensure that the model is generalizing well to unseen or complex images.

We presented a comprehensive report, including a confusion matrix that is a valuable report providing details about the incorrectly classified outcomes of the model. This matrix gives honest and strong insight of the model’s performance by pointing out the misclassified images. It is a table that summarizes the performance of a classification model by displaying the correctly and incorrectly classified instances. The table is typically divided into four cells that constitute the matrix. True positive (TP) represents the number of correct positive predictions, false positive (FP) is a type I error which occurs when the model predicted ‘the occurrence of a condition or an event, but there was no actual occurrence’. Type II error or the false negative cell represents the nonoccurrence of a condition or an event, but there was an actual occurrence. The other two scenarios are true negative outcomes and false negative outcomes. In the case of our study of identifying brain tumors using Vision Transformer, the confusion matrix shows how well the model can classify images into four categories of tumors.

Out of 221 glioma tumor images, 218 were correctly identified and were among true positives. Out of 195 no tumor images, 185 were correctly identified. In the case of meningioma tumor, out of 216 images, 215 were correctly identified. Among the 225 images of pituitary tumor, 223 were correctly identified by the FT-ViT. However, the model also misclassified a small number of images, as shown in the confusion matrix. For example, the model misclassified two pituitary tumor images as meningioma tumor and seven no tumor images as meningioma tumor. Similarly, the model misclassified two no tumor images as glioma tumor, one no tumor image as pituitary tumor, and one glioma tumor image as no tumor. The model also misclassified two glioma tumor images as meningioma tumor and one meningioma tumor image as no tumor.

A classification report was further generated to provide detailed analysis of the proposed model’s performance. In the classification report, the precision scores in terms of probability, the F1 scores in terms of probability, the recall probability, and the accuracy scores for all the four above-mentioned types of brain tumors are provided. For the glioma tumor class, the classification report shows that the model obtained a precision score or value of 0.99 indicating that 99% of the images predicted as glioma tumor were indeed glioma tumor images. The recall score of 0.99 suggests that the model correctly identified 99% of the actual glioma tumor images in the dataset. The F1 score, which balances precision and recall, also reached an impressive value of 0.99. Support 221 means that there are 221 actual glioma tumor images in the dataset. For the meningioma tumor class, the classification table shows that the model obtained a precision score of 0.99, 0.95 recall probability, and F1 score of 0.97. This means that out of all the images predicted as meningioma tumor, 99% were meningioma tumor, and the model was able to correctly identify 95% of all actual meningioma tumor images in the dataset. Support 195 means that there are 195 actual meningioma tumor images in the dataset. For the no tumor class, the report shows that the FT-ViT has a precision value of 0.95, recall probability of 1.00, and F1 score of 0.97. This means that out of all the images predicted as no tumor, 95% were no tumor, and the model was able to correctly identify all actual no tumor images in the dataset. Support 216 means that there are 216 actual no tumor images in the dataset. For the pituitary tumor class, the report shows that the model obtained a precision score of 1.00, 0.99 recall probability, and F1 score of 0.99. This means that out of all the images predicted as pituitary tumor, 100% were pituitary tumors, and the model was able to correctly identify 99% of all actual pituitary tumor images in the dataset. Support 225 means that there are 225 actual pituitary tumor images in the dataset. Overall, these metrics helped us conclude that for all four classes the model can correctly classify brain tumor images with high efficiency, accuracy, and reliability. However, it is important to consider the overall performance of the model and consider factors such as data quality, model complexity, and the choice of evaluation metrics when interpreting the results.

The classification report (Table 1) is a useful tool for evaluating how well the model is working in terms of various evaluation metrics. In this report, the model’s accuracy is given as 0.98, which means that the proposed approach or model correctly classified 98% of the images in the test set. Support 857 means that there are 857 images selected in the validating dataset. The macro average and weighted average of all three scores is 0.98, which indicates that the model performs equally well across all classes. Comparing the results to other methods, we concluded that the FT-ViT model outperformed and gave accuracy of 98.13%.

## 5. Conclusions

In conclusion, the fine-tuned vision transformer model for identifying brain tumors has proven to be a highly accurate and effective method. The achieved accuracy of 98.13% is a testament to the potential of deep learning in the medical field. The model was processed under a series of steps starting from splitting the input into patches, flattening of patches, embedding procedures, concatenation following positional embedding, layer normalization, and a network of feed-forward mechanisms that leads to fine-tuning. The accuracy and loss graph demonstrated that the model efficiently deals with unseen and complex data and features. The outcome was evaluated using accuracy metric, recall probability, precision, and f1-score. The success of this study suggests that fine-tuning pretrained models can be a useful approach for medical image analysis. Further research could help explore the applications of FT-ViT in other fields of the medical imaging process. Future directions can help find ways to improve the interpretability of the model’s predictions and the outcome. Overall, the study provides a promising step towards developing automated and accurate identification and diagnosis systems for various brain tumors.

## Figures and Tables

**Table 1:** The classification report: Evaluating model performance in tumor classification.

	Precision	Recall	F1-Score	Support
Glioma_tumor	0.99	0.99	0.99	221
Meningioma_tumor	0.99	0.95	0.97	195
No_tumor	0.95	1.00	0.97	216
Pituitary_tumor	1.00	0.99	0.99	225
Accuracy			0.98	857
Macro avg	0.98	0.98	0.98	857
Weighted avg	0.98	0.98	0.98	857
Accuracy of the Model: 98.13%				

**Table 2:** Comparative analysis: assessing model performance against alternative methods.

Article	Method	Accuracy
[33]	CNN-SVM-kNN	97%
[34]	GAN	96.25%
[35]	MANet	97.71%
[36]	GAN	96%
[37]	BW-VGG19	98%
Our Study	FT-ViT	98.13%

## Data Availability

Not applicable.
